# Attitudes of Psychiatric Hospital Staff Toward the Risk of Criminal Behavior in Individuals with Mental Disorders

**DOI:** 10.1192/j.eurpsy.2025.1637

**Published:** 2025-08-26

**Authors:** I. Sohn, I. Cho

**Affiliations:** 1 Psychiatry, Keyo Hospital, Uiwang; 2 Psychiatry, WIM psychiatric clinic, SeongNam, Korea, Republic Of

## Abstract

**Introduction:**

Recently, there has been an increase in media reports regarding crimes committed by individuals with mental disorders, leading to a deterioration in public opinion on this issue. Misconceptions about the dangerousness of individuals with mental disorders can negatively impact the prevention, treatment, and social reintegration of these patients.

**Objectives:**

Public attitudes toward crimes committed by individuals with mental disorders are influenced by media and public opinion, and psychiatric hospital staff are not exempt from these influences. Since the prejudices of these staff members can directly affect psychiatric patients, it is crucial to assess their attitudes.

**Methods:**

This study surveyed the attitudes of psychiatric hospital staff regarding the risk of criminal behavior in individuals with mental disorders and compared these attitudes with those of the general population.

**Results:**

The findings revealed that psychiatric hospital staff exhibited less prejudice than the general population across six dimensions related to crimes by individuals with mental disorders: recent increase in crime, cruelty, impulsivity, violence, criminal tendency, and crime rate. Additionally, psychiatric hospital staff displayed less prejudice regarding specific disorders (schizophrenia, depression, bipolar disorder, panic disorder, post-traumatic stress disorder, dementia, attention-deficit/hyperactivity disorder, intellectual disability, and developmental disorders) compared to the general population.

**Conclusions:**

Psychiatric hospital staff demonstrated less prejudice toward the criminal behavior of individuals with mental disorders than the general public. This difference may be attributed to their direct contact with psychiatric patients. The findings suggest potential directions for policy development aimed at reducing public prejudice toward mental disorders and associated criminal behavior.

**Disclosure of Interest:**

None Declared

